# Diffusion dynamics controlled colloidal synthesis of highly monodisperse InAs nanocrystals

**DOI:** 10.1038/s41467-021-23259-w

**Published:** 2021-05-21

**Authors:** Taewan Kim, Seongmin Park, Sohee Jeong

**Affiliations:** Department of Energy Science (DOES) and Center for Artificial Atoms, Sungkyunkwan University (SKKU), Suwon, Gyeonggi-do South Korea

**Keywords:** Quantum dots, Nanoparticles, Synthesis and processing

## Abstract

Highly monodisperse colloidal InAs quantum dots (QDs) with superior optoelectronic properties are promising candidates for various applications, including infrared photodetectors and photovoltaics. Recently, a synthetic process involving continuous injection has been introduced to synthesize uniformly sized InAs QDs. Still, synthetic efforts to increase the particle size of over 5 nm often suffer from growth suppression. Secondary nucleation or interparticle ripening during the growth accompanies the inhomogeneity in size as well. In this study, we propose a growth model for the continuous synthetic processing of colloidal InAs QDs based on molecular diffusion. The experimentally validated model demonstrates how precursor solution injection reduces monomer flux, limiting particle growth during synthesis. As predicted by our model, we control the diffusion dynamics by tuning reaction volume, precursor concentration, and injection rate of precursor. Through diffusion-dynamics-control in the continuous process, we synthesize the InAs QDs with a size over 9.0-nm (1S_max_ of 1600 nm) with a narrow size distribution (12.2%). Diffusion-dynamics-controlled synthesis presented in this study effectively manages the monomer flux and thus overcome monomer-reactivity-originating size limit of nanocrystal growth in solution.

## Abstract (web summary)

Monodisperse colloidal InAs quantum dots have been envisioned as Pb-free materials for various infrared applications. Here, the authors provide a growth model based on monomer diffusion dynamics, enabling extended spectral coverage of InAs quantum dots beyond 1*S*_max_ of 1600 nm.

## Introduction

Colloidal quantum dots (QDs) are materials synthesized using wet chemistry, whose optical and physical properties are controlled by their size and shape due to quantum confinement effects. In particular, the freestanding colloidal QDs allow solution-based chemical post-processing and solution processable thin-film assembly^1,2^. As a result, colloidal QDs have attracted attention in various optoelectronic fields such as bio-imaging, displays, flexible devices, and stretchable devices^3–6^. Chalcogenide colloidal QDs such as PbS, CdSe have been successfully applied to many fields as their synthetic methods were extensively developed, providing a targeted wavelength with high size uniformity^7–11^. Meanwhile, colloidal synthesis of rather covalent colloidal QDs is lagging behind because of difficulties originating from limited control over precursor reactivity in solution^2,12^.

One of the pioneering process designs commonly used to achieve monodisperse colloidal QDs is the hot-injection method^13–15^. Rapid precursor injection provides a burst of supersaturation of monomers, leading to homogeneous nucleation^16^. Further, to induce size-focusing, the growth of QDs should be controlled by monomer diffusion (diffusion-dependent growth) rather than surface reaction (reaction-dependent growth)^17,18^. Diffusion-dependent growth in the hot-injection method can be achieved by controlling monomer reactivity. This growth strategy was successfully implemented in the various particle sizes of the II–VI and IV–VI systems^19,20^. Because both nucleation and subsequent growth are dominated by the supply of a single monomer, hot-injection requires an additional termination step once a specific particle size is achieved, resulting in a residual amount of unreacted monomers, which can induce poor reaction yield ^21^.

On the other hand, the synthesis of colloidal InAs QDs, one of the covalent III–V groups with infrared band gaps, is more challenging. The dehalosilylation of tris(trimethylsilyl)arsine ((TMS)_3_As) has been widely reported in InAs QD synthesis^22–26^. Fast depletion of arsenic precursors during synthesis limits the extension of QD growth. As a result, the growth of large-sized InAs QDs is extremely challenging. InAs QDs of sizes under 1*S*_max_ at 1100 nm have been reported using hot-injection synthesis^24^. Very recently, less reactive aminoarsine precursor-based synthesis has been attempted with a limited half-width-half-maximum (HWHM) of 130 meV with a 1*S*_max_ peak of 920 nm^27^. Because it is evident that control over the chemical kinetics itself cannot provide highly uniform InAs QDs with a 1*S*_max_ peak of 1200 nm, it is necessary to find a new process development solution for their synthesis.

Recently, a continuous precursor injection process has been suggested for synthesizing monodisperse colloidal InAs QDs by exercising strict control of the monomer concentration (*C*_*m*_)^28^. Unlike hot-injection synthesis, where the particle size control can only be achieved during the kinetic growth leading to reduced chemical yield, a continuous precursor injection process can result in a high chemical yield with adequate process design^29^. In 2016, Franke et al. succeeded in preparing monodisperse InAs QDs which have a 1*S*_max_ peak at 1200 nm with 92 meV of HWHM, using a continuous arsenic source injection^30^. In the same year, Tamang et al. introduced firstly clusters as a single-source precursor of InAs QDs, successfully realizing a diffusion-dependent growth mode, thus extending the focusing regime up to 1100 nm of absorption with under 70 meV of HWHM^31^. Understanding the growth process analytically paves the way to redesign the process to extend the spectral coverage of colloidal InAs QDs.

In this study, we investigate a continuous injection process of monodisperse colloidal InAs QD growth by modeling and experimentation. To monitor the growth behavior of InAs QDs as their sizes increased, we compare the volume of QDs and the reactants quantitatively, then derive a growth model of the continuous injection process by mathematical analysis using a modified Fick’s law. Based on the model and experimental data, we find that the concentration gradient between the solution and QDs determines the size range of the InAs QDs and that more optimized synthetic conditions can be provided by controlling the monomer flux. We further design the synthetic process using a diffusion-dynamics-control (DDC) method which provides effective control over monomer flux to extend the growth limit. As a result, we successfully synthesize large-sized InAs QDs with 1600 nm absorption.

## Results and discussion

### Growth behavior of InAs QDs at various precursor injection rates

**Fig. 1:**
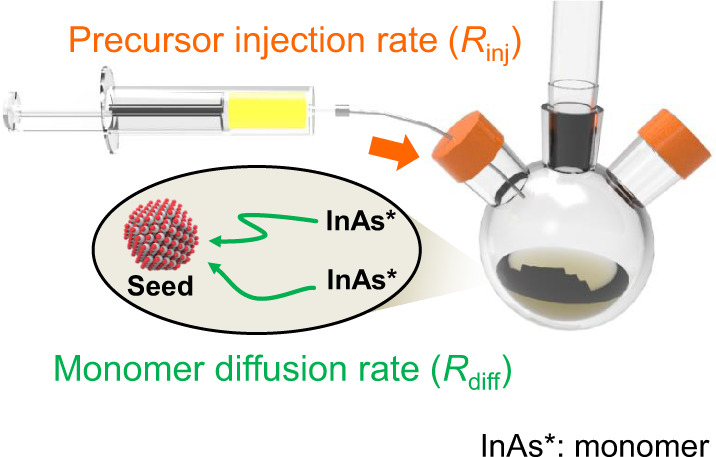
Colloidal InAs QD growth via continuous injection of InAs cluster-based single-source precursor into InAs seed solution. Growth rate is dominated by precursor injection rate (*R*_inj_) and monomer diffusion rate (*R*_diff_).

Figure 1 describes the reaction of colloidal InAs QD growth in the continuous injection process^31^. First, a small InAs QD with a radius of 1.4 nm (seed) is synthesized using the hot-injection method. Then, the InAs cluster-based single-source precursor is slowly added dropwise to the seed solution. The precursor is then converted to a monomer (InAs*) which reacts with the seed in the solution. Here, we assume that if the precursor injection rate (*R*_inj_) is slower than the precursor conversion rate, *C*_*m*_ is always controlled by *R*_inj_ and the monomer diffusion rate (*R*_diff_), as shown in Fig. 1. To investigate the growth behavior as the reaction progressed, we monitored the growth and uniformity of InAs QDs synthesized at different *R*_inj_ values.

**Fig. 2:**
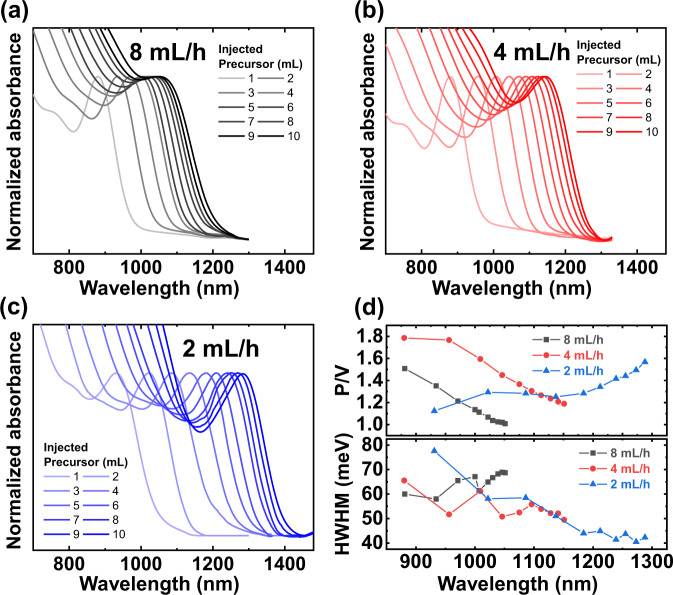
Growth monitoring of colloidal InAs QDs. Normalized absorption spectra of aliquots taken from crude solution were obtained using various precursor injection rates, *R*_inj_: (**a**) 8 mL/h, (**b**) 4 mL/h, and (**c**) 2 mL/h after each 1 mL precursor injection. **d** Peak-to-valley ratio (*P*/*V*) and half-wavelength-half-maximum (HWHM) of the 1st excitonic peaks of (**a**), (**b**), and (**c**).

Figure 2a–c show the absorption spectra of colloidal InAs QDs with different *R*_inj_ values. Equivolume aliquots were taken to measure absorbance at regular intervals. At all *R*_inj_ values, the InAs QD growth became saturated. The 1st excitonic peaks at 8 mL/h and 4 mL/h of *R*_inj_ conditions were observed at 1060 and 1152 nm, respectively, whereas an *R*_inj_ value of 2 mL/h showed 1300 nm, when 10 mL of precursor were injected into each seed solution sample. To investigate the size uniformity of InAs QDs, we observed the peak-to-valley ratios (*P*/*V*) and HWHM in Fig. 2a–c^32–34^. Figure 2d shows that the *P*/*V* values decreased when *R*_inj_ was 8 mL/h or 4 mL/h (black and red lines); for 8 mL/h, the HWHM was enlarged. This led to the conclusion that the growth behavior at a fast *R*_inj_ can disturb diffusion-dependent growth in the continuous precursor injection process.

In contrast, when *R*_inj_ was 2 mL/h (blue line), there was an increase in P/V and narrower HWHM as the size increased, meaning a size-focusing growth of colloidal InAs QDs was obtained. It was observed that *C*_*m*_ should be maintained between specific conditions for size-focusing and to prevent secondary nucleation or interparticle ripening events during the continuous process. A slow *R*_inj_ can suppress burst precursor conversion events to reduce the monomer supply and maintain diffusion-controlled growth^35^. Still, we could not achieve a growth beyond 7 nm (1*S*_max_ = 1300 nm) regardless of additional reaction time and additional precursor injection (Supplementary Fig. 1).

### Quantitative analysis and mathematical modeling using modified Fick’s law

**Fig. 3:**
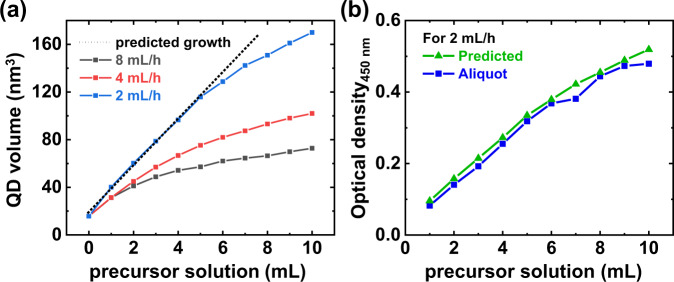
Growth suppression behavior of colloidal InAs QDs. **a** QD volume of Fig. 2a–c (colored line + symbol) and expected QD growth from an amount of injected precursor (black dotted line). **b** Optical density (OD) measured in aliquots of crude solution at 2 mL/h *R*_inj_ at 450 nm (blue) and predicted OD calculated from reaction mixture (green).

According to the growth behavior displayed in Fig. 2, depending on the *R*_inj_ value, the starting point of slowed growth varied significantly. If all the injected monomers were to react completely with the InAs seeds, the volume growth rate of the QD would be directly proportional to the number of monomers being injected, following the ideal growth line (Fig. 3a, dotted line). We then converted our experimental results from Fig. 2 to obtain QD radii from each of the 1*S*_max_ peaks using the Brus equation^36^. Figure 3a shows a quantitative comparison between the experimental results and ideal growth.

Colloidal InAs QDs failed to follow ideal growth at 8 mL/h and 4 mL/h *R*_inj_ conditions (black and red lines in Fig. 3a), implying that unreacted monomers still present. These monomers may cause secondary nucleation and growth instead of contributing to the main particle growth process, reducing the monodispersity of the InAs QDs (see P/V and HWHM values in Fig. 2d). However, for the 2 mL/h *R*_inj_, the size of InAs QDs followed the predicted growth until the precursor volume reach 5 mL (blue line), providing almost 100% reaction between the particles and monomers. Still, the 2 mL/h *R*_inj_ displayed growth suppression after 5 mL of injected precursor.

Furthermore, we investigated the changes in the precursor conversion rate during the synthesis, which are intentionally neglected in Fig. 1, but could affect the colloidal QD growth. First, optical density (OD) at the 2 mL/h *R*_inj_ at 450 nm was measured from the absorption spectrum of the aliquots (Fig. 3b, blue line) and compared to the ideally predicted OD obtained from mathematical calculations (green line). The detailed calculation processes are described in the Methods section. While the size of InAs QDs did not follow the predicted growth in Fig. 3a, the measured and predicted ODs revealed similar values. Because the OD of the cluster precursor is much lower than that of the QD at 450 nm (Supplementary Fig. 2), the measured OD does not contain any contribution from cluster precursors. Therefore, we can conclude that all injected precursors turned into active species e.g., monomers and QDs which absorb photon at 450 nm. Therefore, there is no need to consider the change in precursor conversion when investigating the growth suppression of InAs QDs in this process.

Based on observation on the growth behavior of colloidal InAs QD depending on *R*_inj_, we created a growth model of a continuous precursor injection process. Unlike the hot-injection method, the *C*_*m*_ and reaction volume constantly change in continuous precursor injection, and this change could affect the *R*_diff_. Therefore, we revisited Fick’s law to model diffusion-dependent growth in a continuous process. According to Fick’s first law, the diffusion flux from the solution to the particle surface is proportional to the concentration gradient. Therefore, the particle growth rate in the diffusion-dependent mode can be written as follows^37^:

1 $$\frac{{dr}}{{dt}}\,=\,D\left({C}_{S}\,-\,{C}_{P}\right){V}\!_{m}\frac{1}{r}$$

where *r* is the radius of the particle, *t* is time; *D* is the diffusion coefficient; *C*_*S*_ and *C*_*P*_ are *C*_*m*_ in the solution and particle surface, respectively; and *V*_*m*_ is the molar volume of the material. In a closed system reactor with a fixed solution volume, the interparticle distance is fixed so that the diffusion flux is established in terms of *C*_*S*_. For example, if *C*_*S*_ is sufficiently high compared to the consumed monomer for particle growth, Eq. (1) can be simplified as follows:

2 $$r\,=\,{\left({r}_{0}^{2}\,+\,2D\left({C}_{S}\,-\,{C}_{P}\right){V}_{m}\left(t\,-\,{t}_{0}\right)\right)}^{0.5}$$

where *r*_0_ is the initial particle radius and *t*_0_ is the initial time.

However, Eq. (1) should be considered more carefully in a continuous process. To match the continuous injection process to Fick’s law, we made the following assumptions;

(i) *C*_*P*_ was almost zero and only the change in *C*_*S*_ was reflected in the reaction because the reaction of QD growth is commonly regarded as irreversible^38–41^. In addition, all monomers were assumed to be identical.
(ii) In the modeling, the number of existing monomers in solution (*m*_*j*_) was set constant regardless of the value of *R*_inj_ (1) to simplify the calculation and (2) to design the reaction condition as such that the rate of precursor introduction is slower than the growth rate based on experimental results in Fig. 2. After deriving the model with a fixed *m*_*j*_, we substituted “{*R*_inj_}⋅*t*” for “*t*” to reflect the various precursor injection rate, *R*_inj_, where {*R*_inj_} is the unitless number of *R*_inj_. Additionally, we exclude secondary nucleation events in our model because we designed the *C*_*m*_ below the nucleation threshold.
(iii) We ignored the volume of QD when the distance between the monomers and particles was calculated. Thus, we assumed that only the solvent volume determines the distance.

Under these assumptions, two modifications can be made in Eq. (1). First, *C*_*S*_ is represented by the number of monomers, and the reaction volume increases over time:

3 $${C}_{S}\,=\,\frac{{m}_{j}}{({V}_{i}\,+\,{R}_{{inj}}t)}$$

where *V*_*i*_ is the initial volume of solution. Therefore, the concentration decreases as the reaction occurs. Second, the initial distance is proportional to the one-third power of *V*_*i*_, and the total volume increases as the precursor are injected. Therefore, Eq. (1) can be written as follows:

4 $$\frac{{dr}}{{dt}}\,=\,D\frac{{m}_{j}}{({V}_{i}\,+\,{R}_{{inj}}t)}{V}_{m}\frac{1}{r{\left(1\,+\,\frac{{R}_{{inj}}}{{V}_{i}}t\right)}^{\frac{1}{3}}}$$

Using Eq. (4) and assumption (ii), the particle radius in a continuous process can be calculated as follows:

5 $$r\, =\,{\left({{r}_{0}}^{2}\,+\,\frac{6D{m}_{j}{V}_{m}}{{R}_{{inj}}}\left(\frac{1}{\root{3} \of {\frac{{R}_{{inj}}}{{V}_{i}}{\{{R}_{{inj}}\}t}_{0}\,+\,1}}-\frac{1}{\root{3} \of {{\frac{{R}_{{inj}}}{{V}_{i}}\{{R}_{{inj}}\}t\,+\,1}}}\right)\right)}^{0.5}..\\ =\,{\left({{r}_{0}}^{2}\,+\,\frac{k}{{R}_{{inj}}}\left(\frac{1}{\root{3} \of {\frac{{R}_{{inj}}}{{V}_{i}}{{\{{R}_{{inj}}\}}t}_{0}\,+\,1}}\,-\,\frac{1}{\root{3} \of {\frac{{R}_{{inj}}}{{V}_{i}}{{\{{R}_{{inj}}\}}t}\,+\,1}}\right)\right)}^{0.5}$$

where *k* = 6*Dm*_*j*_*V*_*m*_. Compared to Eq. (2), Eq. (5) shows suppressed growth due to the reduced diffusion rate, and the radius of the InAs QD will be saturated for *t* = infinity. In this model, *t*_0_ and *r*_0_ represent the starting time and initial QD radius in the continuous precursor injection process, respectively.

**Fig. 4:**
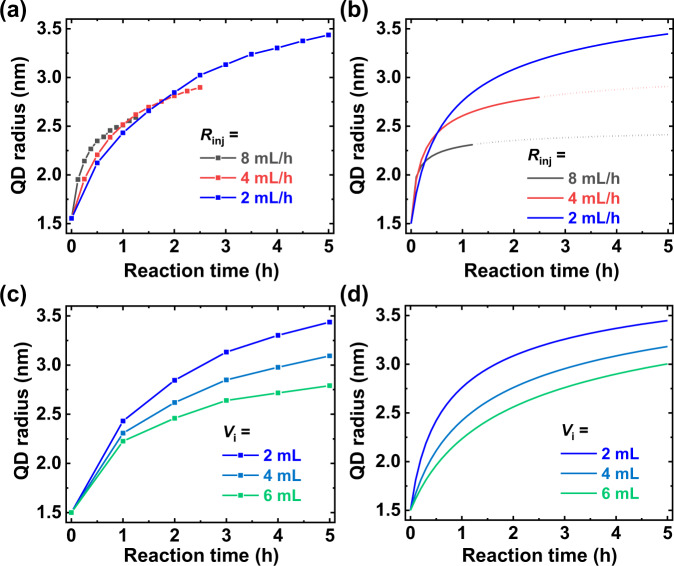
Growth modeling of colloidal InAs QDs. **a**, **b** Radius of InAs QDs at various injection rates (*R*_inj_) for experimental data (**a**) and modeling (**b**). In (**b**), the lines represent the growth for 10 mL volume of injected precursor solution and dotted lines represent further expected growth. **c**, **d** Radius of InAs QD for various initial volumes of solution (*V*_i_) for experimental data (**c**) and modeling (**d**). All experimental conditions and modeling parameters except *R*_inj_ or *V*_i_ are fixed.

To validate our modified model, we compared the experimental and calculated results for the same reaction parameters. Figure 4a, b shows the increase in colloidal InAs QD radius with different values of *R*_inj_ of 8, 4, and 2 mL/h. In both graphs, faster *R*_inj_ reveals a faster increase in radius at the beginning, but as the precursor injection continues, a slower *R*_inj_ results in larger InAs QD size than in the case of faster *R*_inj_. Compared to modeling, the initial growth rate at 8 mL/h was faster, leading to a larger QD radius than the calculated result. This may be due to an increase in *C*_*m*_ because of the remained monomers. Nevertheless, it is predicted that a faster *R*_inj_ induces a burst increase in reaction volume, resulting in growth limitation of InAs QDs.

Furthermore, to investigate the effect of reaction volume, we synthesized colloidal InAs QDs with various *V*_*i*_ values of 2, 4, and 6 mL with a fixed number of seeds (Fig. 4c). As *V*_*i*_ increases, the growth rate of InAs QDs decreases. This growth tendency was obtained by modeling (Fig. 4d), proving that the reduced growth rate originates from the increased reaction volume. After validating our model, we conclude that the growth rate is determined by the monomer concentration gradient.

**Fig. 5:**
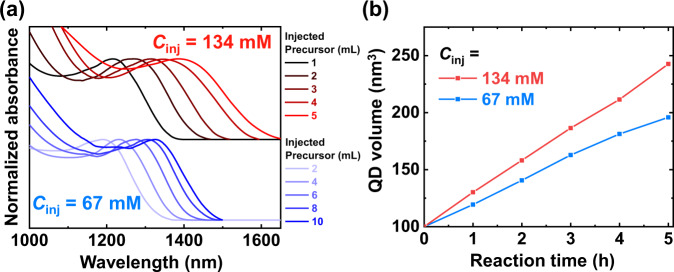
Effect of precursor concentration (*C*_inj_) of injected solution on colloidal InAs QD growth. **a** Normalized absorption spectra of original synthesis condition (*C*_inj_ = 67 mM, blue lines) and concentrated condition (*C*_inj_ = 134 mM, red lines). **b** QD volume derived from (**a**) according to the reaction time. In the concentrated condition, the *C*_inj_ was doubled and the *R*_inj_ was reduced by half to equal the amount of injected precursor per hour.

Based on the model, we optimized the reaction conditions by minimizing the total reaction volume. To diminish the injected volume from 10 mL to 5 mL, the precursor solution was first concentrated from 67 to 134 mM. The OD at 350 nm of the concentrated precursor solution was twice that of the original precursor solution (Supplementary Fig. 3). At this increased precursor concentration, InAs QD synthesis resulted in excessive secondary nucleation (Supplementary Fig. 4). To reduce the additional nucleation, we increased the *V*_*i*_ from 2 to 4 mL, matching the percent increase in precursor concentration. Figure 5 shows the absorption spectra and QD volumes as the precursor was injected. Herein, *R*_inj_ was reduced by half to equal the amount of injected precursor per hour.

As the total reaction volume decreased from 12 to 9 mL, the size of colloidal InAs QDs based on the 134 mM precursor solution exhibited its 1st excitonic peak at 1400 nm, while InAs QDs based on 67 mM had its peak at 1330 nm with the same amount of injected precursor. Moreover, the 134 mM precursor solution exhibited a higher slope and more linear growth than at 67 mM (Fig. 5b). This result indicates that the optimal condition for reducing the total reaction volume was effective for InAs QD growth. Nevertheless, we cannot keep decreasing the total reaction volume mainly due to the secondary nucleation event. Thus, to overcome the current growth limit of monodisperse InAs QDs, a new synthetic strategy is required to further reduce the concentration gradient.

### DDC continuous process

From the modified Fick’s law model and experimental data, we can expect that the existing continuous injection process cannot be used to synthesize colloidal InAs QDs with 1st excitonic peaks greater than 1500 nm by optimization of the reaction conditions. Herein, we introduce a simple DDC continuous process to continuously grow InAs QDs via continuous injection.

**Fig. 6:**
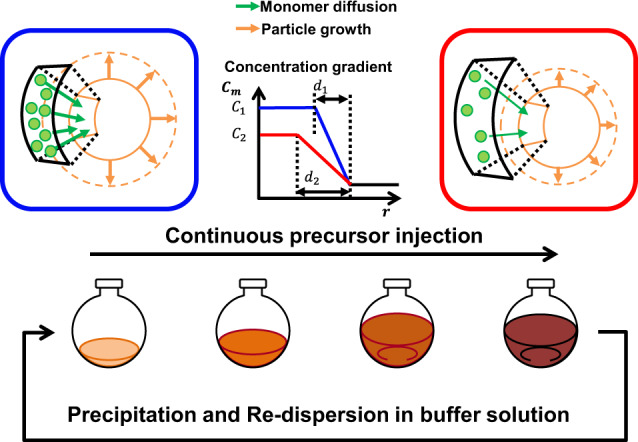
Diffusion-dynamics-controlled (DDC) continuous process. At the beginning of the synthesis, monomers (green circles) diffuse to particles (orange circle) rapidly because of a high *C*_m_ (*C*_1_) and a short diffusion length (*d*_1_). After enough precursors are injected, *C*_m_ decreases (*C*_2_), and the diffusion length increases (*d*_2_), leading to slow particle growth. Thus, precipitation and re-dispersion in the buffer solution with the same volume of initial solution can reset the reduced concentration gradient and improve the growth rate of InAs QDs.

Figure 6 shows the DDC process for colloidal InAs QD growth. At the beginning of the reaction, a high *C*_*m*_ (*C*_1_) and short diffusion path (*d*_1_) lead to the fast growth of InAs QDs. However, as the solution volume increases, *C*_*m*_ decreases (*C*_2_), and the diffusion path is increased (*d*_2_); therefore, the diffusion rate of the InAs QD becomes suppressed during the synthesis. To recover the reduced diffusion rate, we purified the InAs QD crude solution by centrifugation and re-dispersed the precipitate in a volume of buffer solution equal to the initial seed solution volume (2 mL). The buffer solution contained In-oleate to stabilize the InAs QDs (see the Methods section for more details).

**Fig. 7:**
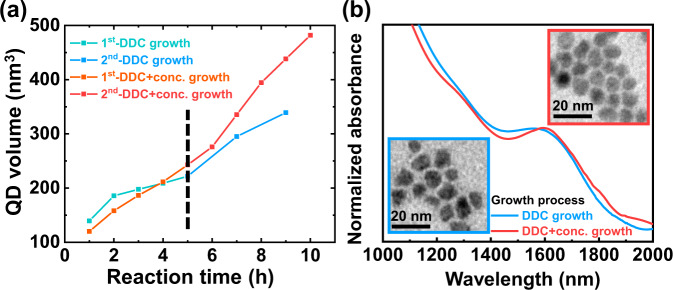
Growth of colloidal InAs QDs using the DDC process. **a** InAs QD growth using twice of DDC processes with (orange and red) and without concentration control (cyan and blue) of precursors. **b** Absorption spectra of InAs QDs grown using DDC for 9 h showing 1st excitonic peaks at 1579 (blue line) and using DDC with concentration control for 10 h showing 1600 nm (red line), respectively. Inset: Transmission electron microscope (TEM) images of InAs QDs.

Figure 7a shows the colloidal InAs QD volume increase for the injected precursor at various *R*_inj_ values via the DDC process. InAs QDs with around 1300 nm of 1st excitonic peak were used as seeds for DDC processes. Each time the DDC process was performed, an increase in the growth rate of InAs QD volume was observed, indicating that the reduced distance between monomers and QDs using the decreased reaction volume accelerated monomer diffusion dynamics. Furthermore, to enhance the size range and uniformity of InAs QDs, we optimized the DDC process by controlling the precursor concentration as suggested in Fig. 5 (see the Methods section for more details). Same to synthesis in Fig. 5, the amount of injected precursor per hour in both reactions was equal. The controlling of precursor concentration provides a more linear growth tendency of InAs QDs compared to the increase of InAs QD volume without the control. The sizes of InAs QDs with various reaction times were well matched with the reported Brus equation (Supplementary Fig. 5)^36^.

At the end of the twice of DDC process, the resulting InAs QD shows absorption spectra with a 1st excitonic peak at 1579 nm, a *P*/*V* value of 1.03, and a HWHM of 83.5 meV. Further optimizing DDC processes by increasing the precursor concentration from 67 mM to 134 mM provided InAs QDs with a 1st excitonic peak at 1600 nm displaying a *P*/*V* value of 1.12 and a HWHM of 60.5 meV (Fig. 7b). The size of InAs QDs synthesized by both DDC and DDC with concentration control methods are 7.2 and 9 nm with coefficient of variation of 15.0% and 12.2%, respectively (Fig. 7b, inset and Supplementary Fig. 6). The InAs QDs showed a zinc-blende crystal structure in XRD measurement (Supplementary Fig. 7).

We confirmed that the growth of colloidal InAs QDs is largely extended to a larger size regime by effectively managing the monomer flux using the DDC process. The optimized reaction condition also prevents anisotropic growth such as branched or angled shapes (Supplementary Fig. 1 and Fig. 7b (blue edged TEM image)) often induced from thermodynamics-driven reactions in colloidal synthesis^42,43^. Because a decreased reactivity of large QDs further promotes thermodynamically controlled growth, the control of monomer flux becomes more important in the growth of large-size QDs.

In conclusion, we developed a particle growth model for a continuous precursor injection process and investigated the influence of monomer flux on the tunable size range of monodisperse colloidal InAs QDs. The model was verified by comparison with the quantitative analysis of QD growth and modeling was able to provide the InAs QD growth tendency with various reaction parameters. It was shown in the model that although slower precursor injection compared to monomer diffusion induces larger and monodisperse InAs QDs, the monomer flux becomes reduced by the injected solution volume increase during the QD growth, leading to an inversion of precursor injection and monomer diffusion. To control the monomer flux effectively, we designed a DDC process, which adjusts the total reaction volume by removing unnecessary solvents. Tuning of the monomer flux by the DDC process provided monodisperse colloidal InAs QDs showing 1600 nm at the 1st excitonic peak with a 9 nm size, and to the best of our knowledge, this is the first time this has been reported. We believe that the control of monomer flux is essential to synthesize large and monodisperse InAs QDs. Our findings are based on monomer dynamics in colloidal synthesis, enabling an improved understanding of growth behavior regardless of the materials used. The model and process developed here could benefit from designing nanoparticle synthesis aim at various applications.

## Methods

### Materials

Indium(III) acetate (InOAc, UNIAM, 99.999%), oleic acid (OA, UNIAM, ≥99%), 1-octadecene (ODE, UNIAM, 99.999%), and dioctylamine (DOA, Aldrich, ≥97%) were used as purchased without any further purification. Tris(trimethylsilyl)arsine ((TMS)_3_As, JSI silicon, 99%) was distilled before use. All solvents, including hexane, butanol, and tetrachlorethylene (TCE), were purchased from Sigma-Aldrich Chemical Company.

### Continuous injection process of colloidal InAs QDs

Both InAs seeds and InAs clusters were prepared using the same In and As precursor solutions. For the seed solution, 1 mmol InOAc and 3 mmol OA were degassed in 5 mL of ODE (100 °C for 2 h). A measure of 0.5 mmol of TMS_3_As was mixed to 1.5 mmol DOA with 1 mL ODE in a glove box and annealed for 10 min at 60 °C. After degassing, the In precursor solution was heated to 300 °C under nitrogen condition, and the As precursor solution was rapidly injected into the degassed In solution. For the cluster precursor, In and As precursor solutions were prepared using the same method and reacted at 25 °C under nitrogen condition. The amount of cluster was determined fluidly by experimental conditions.

To equal the amount of seed in various experimental conditions, the seed solution was diluted with buffer solution (see DDC process section in details) at room temperature, and this was confirmed by the value of OD through absorption measurement. Total volume of seed solutions was unified to 2 mL and optical densities at 450 nm were 0.156 based on 300 times dilution of the solutions. The seed solution was heated to 300 °C, and the cluster solution was dropped onto the seed using syringe pump at desired injection rates. The synthesized InAs QD crude solution was purified by hexane and butanol 3 times.

To perform the concentrated precursor solution-based growth, two times of the InOAc, OA, TMS_3_As, and DOA were used for the same amount of solvent compared to the initial condition. Then, it was injected into a 4 mL monomer solution that was doubly diluted.

### Diffusion-dynamics-controlled (DDC) process

The buffer solution was prepared using InOAc, OA, and DOA. To balance the chemical equilibrium, one-half of InOAc and OA were used for the buffer solution compared to the seed or cluster solution. Purified InAs QDs were first diluted with hexane, and the mixture of the InAs QD solution and 2 mL of buffer solution was then slowly degassed to remove water, oxygen, and hexane at 100 °C. After the degassing, the solution was heated to 300 °C in 20 min and original or concentrated precursor solution was injected at desired injection rates.

### Characterization of InAs QDs

The absorption spectra were measured using an ultraviolet/visible/near-infrared spectrophotometer (Shimadzu, UV3600). To remove the organic peaks, TCE was used as a solvent. The shape and size of the InAs QD were observed using TEM (Carl Zeiss, LIBRA 120). To investigate the crystal structure of the QDs, XRD was performed using an X-ray diffractometer (Rigaku, SmartLab).

### Growth modeling of InAs QDs

The growth simulation of InAs QDs was calculated by MATLAB program. Initial volume (*V*_*i*_) and injection rate (*R*_inj_) were used as the same values as the experimental values. The initial radius (*r*_0_) of 1.5 nm was used and initial time (*t*_0_) was 0. Proportional constant *k* of 35 was used for all of the modelings to clearly observe the trend of QD growth.

### The ideal size of QD under continuous precursor injection and optical density

The QD concentration of seed solution was calculated by dividing OD at 450 nm of seed solution by molar extinction coefficient and optical path length as below^31^.

6 $${C}_{{seed}}\,=\,\frac{{{OD}}_{450,0}}{{\varepsilon }_{450,0}L}$$

where ${C}_{{seed}}$, ${{OD}}_{450,0}$, ${\varepsilon }_{450,0}$, and $L$ is the QD concentration of seed solution, OD at 450 nm, molar extinction coefficient, and optical path length, respectively. The ${\varepsilon }_{450}$ is 1.59 × 105 *r*^3^ (M^−1^ cm^−1^), where *r* is radius of QD determined by Brus equation with the 1st excitonic peak of solution. Then the number of QDs (${N}_{{QD}}$) is product of ${C}_{{seed}}$ and the volume of seed solution.

To predict ideal growth of QD, increased InAs unit cell by precursor injection in total solution was obtained by multiplying injected amount of As ion and molar volume of InAs as below.

7 $${\triangle V}_{{InAs},{total}}\,=\,{C}_{{precursor}}{V}_{{precursor}}{V}_{m}$$

where ${\triangle V}_{{InAs},{total}}$, ${C}_{{precursor}}$, ${V}_{{precurs}{or}}$, and ${V}_{m}$ is increased InAs unit cell in total solution, the molar concentration of As precursor, volume of the precursor solution, and molar volume of InAs, respectively. Because this synthesis is In-rich reaction, the ideal number of InAs unit cells is the same as the number of As ions.

Then, final volume of a grown QD is obtained as below.

8 $${V}_{{QD}}\,=\,{V}_{{QD},0}\,+\,\frac{{\triangle V}_{{InAs},{total}}}{{N}_{{QD}}}$$

where ${V}_{{QD}}$ is volume of a grown QD and ${V}_{{QD},0}$ is volume of a seed QD determined by Brus equation.

In last, the predicted OD in Fig. 3b was calculated by molar extinction coefficient and the concentration of QD solution

9 $${{OD}}_{450}\,=\,{\varepsilon }_{450}{C}_{{solution}}L\,=\,{\varepsilon }_{450}\frac{{N}_{{QD}}}{{V}_{{solution}}}L$$

where ${V}_{{solution}}$ is volume of the QD solution and ${C}_{{solution}}$ is concentration of solution. Herein, ${\varepsilon }_{450}$ is calculated by expected radius from ${V}_{{QD}}$. The shape of QD is assumed to be a sphere. The ${N}_{{QD}}$ is fixed because there is no secondary nucleation or interparticle ripening in ideal case.

## Supplementary information

Supplementary Information

## Data Availability

The data that support the plots within this paper and other findings of this study are available from the corresponding authors on request.
