# Supplementary material for: Diffusion dynamics controlled colloidal synthesis of highly monodisperse InAs nanocrystals
Source: Nat Commun. 2021 May 21;12:3013. doi: 10.1038/s41467-021-23259-w (PMC8140152; doi:10.1038/s41467-021-23259-w)
Supplement: Supplementary file 1 — Supplementary Information [file 41467_2021_23259_MOESM1_ESM.pdf]

Diffusion dynamics controlled colloidal synthesis of highly monodisperse InAs nanocrystals

Taewan Kim[1,2], Seongmin Park[1,2], and Sohee Jeong[1,*]

[1] Department of Energy Science (DOES) and Center for Artificial Atoms, Sungkyunkwan University (SKKU), Suwon, Gyeonggi-do 16419, South Korea.

[2] These authors contributed equally

Corresponding Author

S. Jeong (s.jeong@skku.edu)

Supplementary Figure 1. Normalized absorption spectra and transmission electron microscopy (TEM) image of growth suppressed InAs QD.

Supplementary Figure 2. Absorption spectra of InAs QD and InAs nanocluster.

Supplementary Figure 3. Absorption spectra of original and doubly concentrated precursor solution.

Supplementary Figure 4. Normalized absorption spectra of the colloidal InAs QDs synthesized with different precursor concentration.

Supplementary Figure 5. Absorption spectra, Brus equation, and TEM images of various InAs QDs and the diameter histograms.

Supplementary Figure 6. Diameter histogram of InAs QDs synthesized by DDC process.

Supplementary Figure 7. X-ray diffraction pattern of InAs QD and reference zinc-blende of InAs.

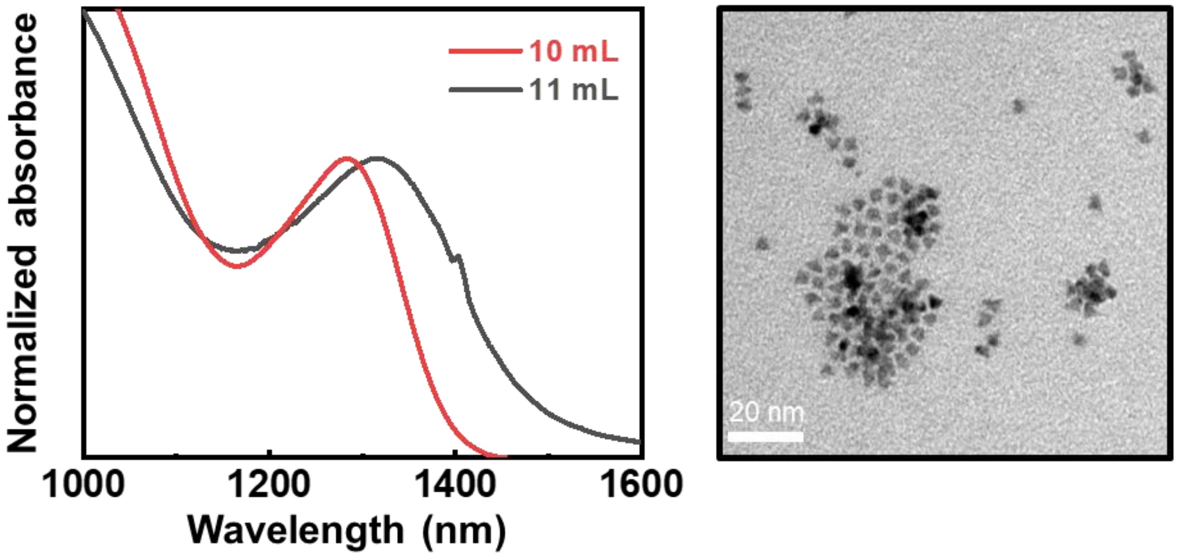

Supplementary Figure 1. Normalized absorption spectra and TEM image of growth suppressed InAs QD produced by 11 mL of precursor injection. Injection rate is 2 mL/h. Additional precursor injection after the growth limit often resulted in anisotropic, branched particle shape of InAs QDs.

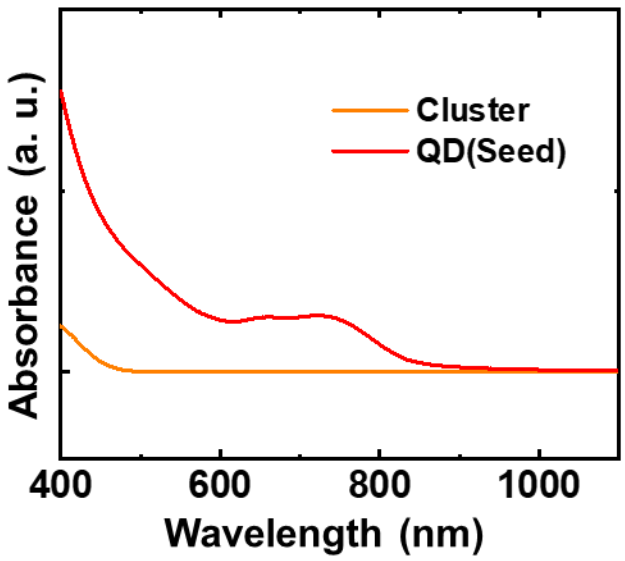

Supplementary Figure 2. Absorption data of InAs QD (seed) and InAs nanocluster prepared using the same molar concentration of In and As sources.

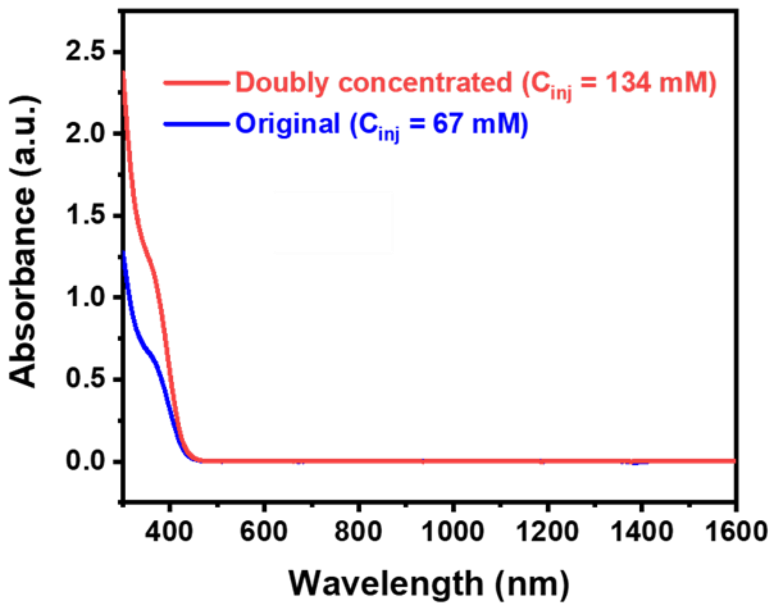

Supplementary Figure 3. Absorption spectra of original (67 mM, blue) and doubly concentrated (134 mM, red) precursor solution in Fig. 4. C_{inj} is precursor concentration of solution.

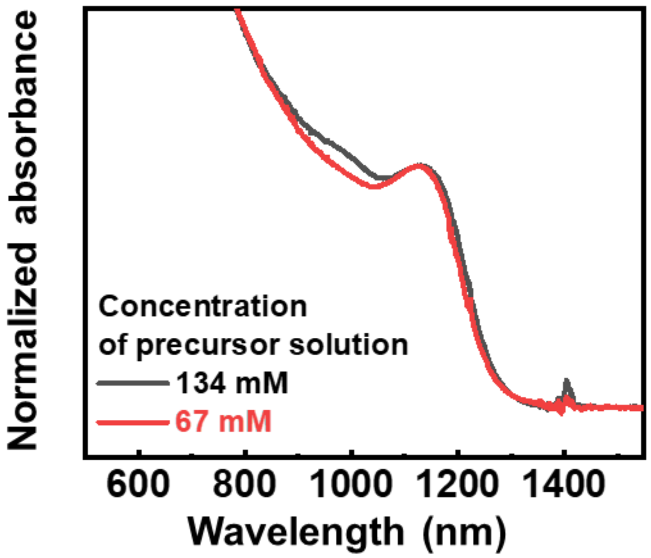

Supplementary Figure 4. Normalized absorption spectra of the colloidal InAs QDs synthesized with 134 mM (black) and 67 mM (red) precursor solution. Additional peak from secondary nucleation occurs at around 950 nm in the case of 134 mM.

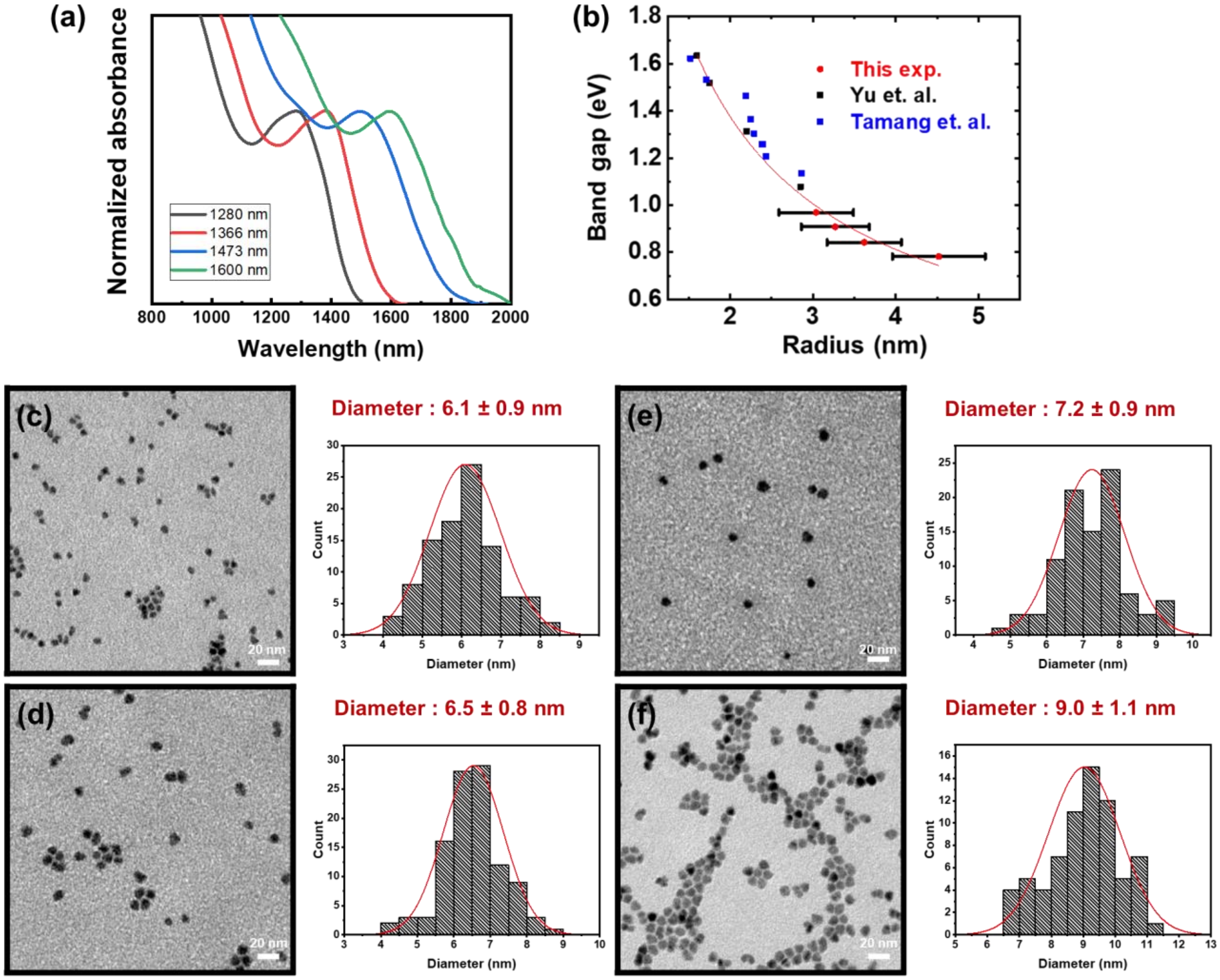

Supplementary Figure 5. Absorption spectra (a), Brus equation reported by literature[1] (b), and TEM images (c-f) of various InAs QDs and the diameter histograms. Literature values[1,2] (black, blue) and experiments in this study (red) were plotted in (b). The black lines behind the red dots indicate the standard deviation of each of the data points. We assumed that the shape of the InAs QDs was spherical, as reported previously. Each of the InAs QDs observed in (b), (c), (d), and (e) reveals 1st excitonic peaks at 1280, 1366, 1473, and 1600 nm, respectively.

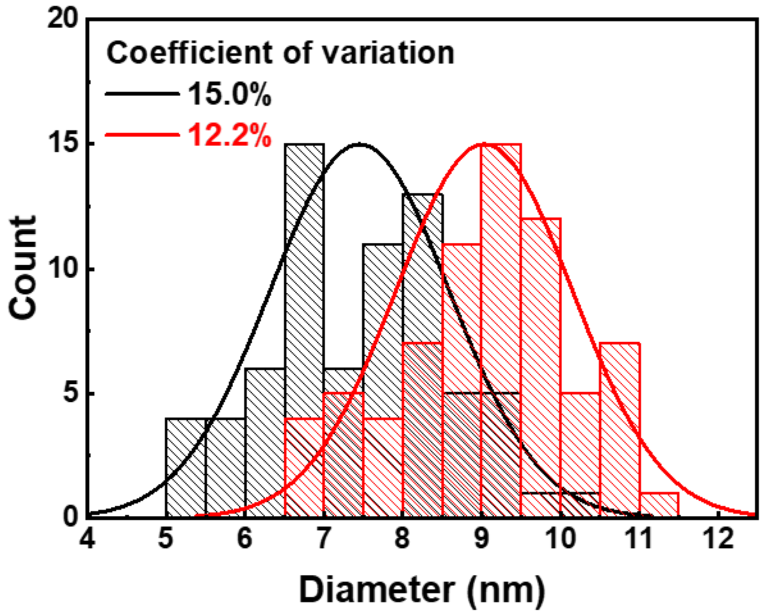

Supplementary Figure 6. Diameter histogram of InAs QDs synthesized by DDC process. Average diameters are 7.2 and 9 nm, respectively.

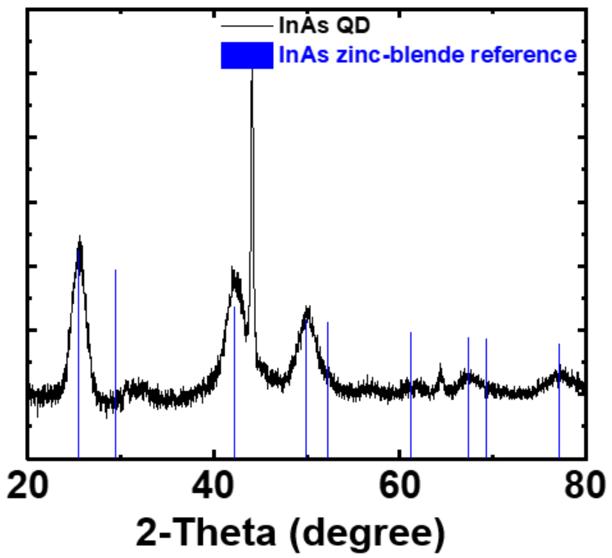

Supplementary Figure 7. X-ray diffraction pattern of InAs QD and reference zinc-blende of InAs. Sharp peak around 44 degree is iron substrate peak of XRD instrument.

1. Yu, P. *et al.* Absorption cross-section and related optical properties of colloidal InAs quantum dots. *J. Phys. Chem. B* **109**, 7084–7087 (2005).

2. TaMang, S., Lee, S., Choi, H. & Jeong, S. Tuning size and size distribution of colloidal InAs nanocrystals via continuous supply of prenucleation clusters on nanocrystal seeds. *Chem. Mater.* **28**, 8119–8122 (2016).
